# Immunosenescence of Natural Killer Cells, Inflammation, and Alzheimer's Disease

**DOI:** 10.1155/2018/3128758

**Published:** 2018-11-01

**Authors:** Corona Solana, Raquel Tarazona, Rafael Solana

**Affiliations:** ^1^Centro Hospitalar Psiquiátrico, Lisbon, Portugal; ^2^Immunology Unit, University of Extremadura, Caceres, Spain; ^3^Instituto Maimónides de Investigación Biomédica (IMIBIC), Córdoba, Spain; ^4^Reina Sofia University Hospital, Córdoba, Spain; ^5^University of Cordoba, Córdoba, Spain

## Abstract

Alzheimer's disease (AD) represents the most common cause of dementia in the elderly. AD is a neurodegenerative disorder characterized by progressive memory loss and cognitive decline. Although the aetiology of AD is not clear, both environmental factors and heritable predisposition may contribute to disease occurrence. In addition, inflammation and immune system alterations have been linked to AD. The prevailing hypothesis as cause of AD is the deposition in the brain of amyloid beta peptides (A*β*). Although A*β* have a role in defending the brain against infections, their accumulation promotes an inflammatory response mediated by microglia and astrocytes. The production of proinflammatory cytokines and other inflammatory mediators such as prostaglandins and complement factors favours the recruitment of peripheral immune cells further promoting neuroinflammation. Age-related inflammation and chronic infection with herpes virus such as cytomegalovirus may also contribute to inflammation in AD patients. Natural killer (NK) cells are innate lymphoid cells involved in host defence against viral infections and tumours. Once activated NK cells secrete cytokines such as IFN-*γ* and TNF-*α* and chemokines and exert cytotoxic activity against target cells. In the elderly, changes in NK cell compartment have been described which may contribute to the lower capacity of elderly individuals to respond to pathogens and tumours. Recently, the role of NK cells in the immunopathogenesis of AD is discussed. Although in AD patients the frequency of NK cells is not affected, a high NK cell response to cytokines has been described together with NK cell dysregulation of signalling pathways which is in part involved in this altered behaviour.

## 1. Introduction

Alzheimer's disease (AD) is the most prevalent form of dementia, characterized by memory loss and cognitive decline, often associated with behavioural disorders [1–4]. According to the World Alzheimer Report 2016 [4], there were 46.8 million people worldwide living with dementia in 2015 and this number will reach 131.5 million in 2050. The most frequent form of AD, often referred to as late onset AD, has a sporadic onset and progress to neurodegeneration over a period of several years and occurs usually after the age of 65. It has also been described an early onset form of AD that appears before the age of 65 probably due to genetic mutations leading to an overproduction of amyloid beta peptides (A*β*) in the patient's brain. In both forms of AD, alterations in the A*β* cascade have been involved in neuronal loss, memory loss, and alterations of other cognitive functions [5, 6].

The amyloid cascade hypothesis states that the accumulation of A*β* in the form of senile plaques, the hyperphosphorylation of the Tau protein, and the subsequent formation of neurofibrillary tangles are the causes of AD. Recently, the neuroinflammation hypothesis supporting that brain inflammation is involved in the development and progression of AD has gained acceptance, although whether inflammation is cause or consequence of the accumulation of A*β* is still unclear [7–13]. Thus, considering AD as a chronic inflammatory disease, a role of the immune system in the development or progression of AD has been proposed [14, 15].

## 2. Alzheimer's Disease

In 1907, Alois Alzheimer described a disease characterized by severe cognitive disturbances, disorientation, aphasia, delusions, and unpredictable behaviour. The disease progressed and the patient died 4.5 years later. He discovered the presence of brain atrophy in the pathological examination and characteristic alterations that nowadays are referred to as neurofibrillary tangles. In 1910, the disease was named after him by Kraepelin receiving the denomination of Alzheimer's disease [16].

Although AD has been historically defined as beginning once dementia symptoms appear, the National Institute on Aging (NIA) and the Alzheimer's Association published in 2011 revised diagnostic guidelines including biomarkers of brain changes [17–19]. Thus, in addition to clinical symptoms, the “A/T/N” system in which “A” refers to the value of a *β*-amyloid biomarker, “T” to the value of a tau biomarker, and “N” to biomarkers of neuronal injury has been incorporated for early diagnose of AD [20]. According to the presence of these biomarkers and clinical symptoms at least three stages of AD have been proposed: preclinical AD, which corresponds to clinically normal individuals but with the presence of some of the biomarkers of brain changes; mild cognitive impairment (MCI) due to AD, a stage characterized by both brain changes and mild cognitive symptoms that do not affect everyday living; and dementia due to AD, a stage with brain changes and significant memory, thinking, and behavioural problems that interfere with an individual's daily life [20–22]. Amnestic MCI (aMCI) can be identified by neuropsychological tests [23] and almost half of the aMCI individuals progress to AD within 3 years [24]. Age is the greatest risk factor of late onset AD, which affects 10% of people older than 65. The percentage of people with AD increases from 3% of people aged 65-74 to 17% of people aged 75-84 and 32% of people aged 85 or older. Therefore, 81% of AD patients are 75 years old or older [25].

The exact cause of AD is still not known although many scientists believe in the beta amyloid hypothesis which states that the accumulation of A*β* in brain is the initial cause which consequently leads to pathological neuroinflammation. In the last few years it has been shown that A*β* may have an important role in defending the brain against infections and the hypothesis that altered immune and inflammatory responses against, still undefined, infectious organisms play a role in the development and progression of AD has been a matter of investigation in recent years [7–13]. Thus, it has been suggested that microbial infection may be involved in AD pathogenesis [26–28]. Thus, neurotropic human herpesviruses (HHV) have been connected with neurodegenerative diseases, including AD, in the context of other stressors and genetic risk factors. The contribution of herpes simplex virus 1 (HSV-1), HHV-6, or cytomegalovirus (CMV) to AD pathogenesis has been proposed by several authors [29–31]. A recent study has shown in three independent cohorts increased HHV-6A and HHV-7 in brain regions from human postmortem tissue in AD patients compared to controls. These authors also links molecular, clinical, and neuropathological features with viral activity, supporting that viral activity constitutes a general feature of AD [32].

Although AD was formerly considered a brain disease; nowadays it is viewed as a systemic disease. The blood brain barrier is compromised in AD allowing migration of peripheral immune cells to the brain and* vice versa*. In addition, altered blood brain barrier allows transport of inflammatory mediators to the circulation activating immune cells and promoting their migration into the brain [15]. Thus, high levels of tumour necrosis factor (TNF)-*α* in sera was associated with a 4-fold increase in the rate of cognitive decline [33]. Pathogen induced inflammation in the periphery may also contribute to the brain inflammation by increasing blood brain barrier permeability that enables the traffic of peripheral immune cells to the brain. Altogether, it has been suggested that the activation status of peripheral innate immune cells represents an early biomarker of brain inflammation in AD patients [15]. Persistent viral infection by herpesvirus such as CMV has been proposed to be, to some extent, responsible of the pathological changes observed in AD. Thus, interaction between CMV and HSV-1 was associated with AD development possibly by affecting the immune system [34].

## 3. Immune System, Inflammation, and Alzheimer Disease

Thus, as mentioned above, the principal hypothesis to explain AD is the deposition of A*β* forming senile plaques that cause AD and generate the other hallmark of the disease such as the neurofibrillary tangles [35–37]. Recently, a role of neuroinflammation in the development and progression of AD has been proposed. Neuroinflammatory responses involve both cellular and molecular players [38]. Amyloid deposits can lead to chronic neuroinflammation, tau hyperphosphorylation and loss of synapses and neurons responsible of brain atrophy and cognitive decline [39]. In addition, microglial activation has been considered to play a pivotal role in the pathogenesis of sporadic forms [40].

Accumulation of A*β* plaques induces the activation of complement system that can lead to neuronal damage and death. Thus, neurons secrete C1q that binds A*β* and activates C1q receptor (C1qR) on microglia promoting phagocytosis of A*β*. In addition, astrocytes are stimulated by inflammatory signals and secrete C3 that is cleaved into C3b and C3a. Complement peptide C3a mediates recruitment of peripheral immune cells to the brain [41].

Neuroinflammatory cascades rely on the activation of microglial NLRP3 inflammasome. It has been shown that A*β* deposits can activate NLRP·inflammasome leading to the production of interleukin (IL)-1*β* and IL-18 that may contribute to the pathogenesis of AD and cause cognitive impairment [42, 43]. It cannot be excluded that A*β* deposits can be also the consequence of inflammasome activation in AD patients. NLRP3 inflammasome activation is restricted to plaque-associated microglia further supporting its role in AD pathogenesis [42].

Several pieces of evidence have demonstrated a critical involvement of innate immune system in the pathogenesis and progression of AD. It has been suggested that A*β* deposits activate microglia by interacting with surface receptors such as Toll like receptors (TLRs). TLRs recognize pathogen associated molecular patterns such as bacterial peptidoglycans and lipopolysaccharide recognized by TLR2 and TLR4, respectively [44]. It has been described that TLR2 and TLR4 also recognize A*β* [45, 46]. Once activated, microglial cells produce proinflammatory cytokines and chemokines. In an early phase of AD, microglia is involved in phagocytosis and clearance of A*β*, however, when AD progresses microglia function is impaired with diminished phagocytic capacity, low TLR4 expression and high production of anti-inflammatory cytokines [47].

Together with inflammasome activation and the production of inflammatory cytokines, cellular components of the immune system such as granulocytes, monocytes, Natural killer (NK) cells, and T cells can also participate in the pathogenesis of neuroinflammation [48, 49]. Thus, changes in both innate and adaptive immune system have been associated with AD. As disease progresses, the immune system is severely affected in AD patients [50] with a decreased frequency and diminished function of T and B cells [51] and disturbed proinflammatory cytokine production [52]. NK cells have also been involved in different brain diseases including AD [53].

The triple transgenic mice for AD (3xTgAD) constitute an experimental model that mimic the human AD pathophysiology. The immune system of these animals shows changes associated with premature immunosenescence at 4 months of age, when the immunoreactivity against intracellular A*β* fibrils appears. In addition, alterations in the percentage and cytotoxic activity of NK cells are observed, at the age of 2 months before the onset of AD, suggesting that changes in peripheral immune cell functions, in particular in NK cells, could be early peripheral markers of the preclinical and prodromal stages of AD [54].

## 4. NK Cells in Healthy Ageing

NK cells are innate lymphoid cells (ILCs) that represent approximately 15% of peripheral blood lymphocytes. NK cells are cytotoxic lymphocytes that share many features with ILC1, such as their capacity to produce interferon (IFN)-*γ*, although they are developmentally distinct [55]. Several NK cell subsets can be distinguished according to the differential expression of some phenotypical and functional markers. NK cells expressing high levels of surface CD56 (CD56^bright^) that represent less than 10% of peripheral blood NK cells are more immature and have an immunomodulatory role with high production of cytokines and chemokines, whereas the major NK cell subset (about 90%) are mature CD56^dim^CD16^+^ NK cells characterized by high cytotoxic capacity and IFN-*γ* production after direct contact with tumour or virus-infected target cells [56, 57]. A model of differentiation from immature CD56^bright^ that leads to more mature CD56^dim^ NK cells has been proposed [58]. Another subpopulation of NK cells, which do not express CD56 but express other NK receptors, was originally described in HIV-infected patients [59]. In addition, the expression of CD57 is considered a marker of highly differentiated NK cells [60]. A model of peripheral blood NK cell maturation proposes a gradual shift from CD56^bright^ via CD56^dim^CD57^−^ to CD56^dim^CD57^+^ NK cells [61–64].

Several cytokines such as IL-12, IL-15 or IL-18 can activate NK cells triggering the production of IFN-*γ* [65]. NK cells also produce other chemokines and cytokines such as IL-10, a cytokine with immunosuppressive functions [66]. In addition, NK cell crosstalk with macrophages and dendritic cells regulates their activation and function [67].

**Figure 1:**
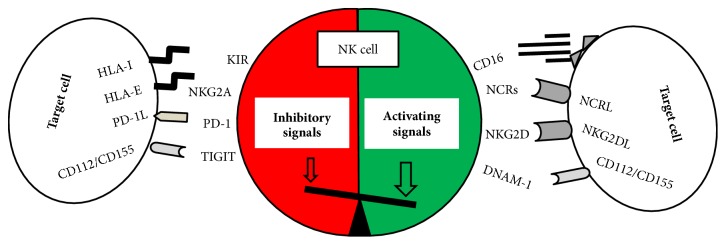
**Balance of inhibitory and activating signals in NK cell activation.** Inhibitory signals are mediated through receptors specific for human leukocyte antigen (HLA) class I molecules and other inhibitory receptors such as PD-1 (ligand PD-1L) and TIGIT (ligands CD155 and CD112). Activating signals are transmitted though several surface receptors that recognize ligands expressed on transformed cells. The balance between inhibitory and activating signals will determine NK cell activation and function.

NK cell function, cytotoxicity and cytokine production, depends on a balance between activating and inhibitory signals triggered by activating and inhibitory receptors (Figure 1) expressed on NK cells [68]. Killer immunoglobulin-like receptors (KIR) and NKG2A are the most important inhibitory receptors recognizing major histocompatibility complex (MHC) class-I molecules. These inhibitory receptors act as sensors of healthy cells protecting them from NK cell-mediated cytotoxicity. Thus, loss of MHC class-I expression is frequently observed on virus infected cells and tumour cells allowing NK cells to recognize those transformed cells. NK cells also express inhibitory receptors recognizing other ligands than MHC class-I molecules. These inhibitory receptors such as PD-1, TIGIT, LAG-3, and TIM-3 represent novel checkpoints for immunotherapeutic strategies against cancer [69]. To the best of our knowledge, the impact of these receptors in AD has not been analysed so far. In addition, NK cells detect stress markers expressed on virus infected and tumour transformed cells through activating receptors [70].

Aging can be defined as the time-dependent functional decline characterized by a progressive loss of anatomic and physiological integrity, leading to impaired function and increased vulnerability to death. Nine cellular and molecular hallmarks of aging have been proposed that are generally considered to contribute to the aging process and define the aging phenotype. Aging is the primary risk factor for major human pathologies including neurodegenerative diseases [71]. In particular, age represents the major risk factor of late onset AD [25].

Immunosenescence refers to the gradual age-associated decline of the immune system that contributes to the increased incidence of infectious diseases and probably to the high incidence of cancer in the elderly. Thus, health status in the elderly is correlated to the immune system and an immune risk phenotype (IRP) has been suggested [72]. Recently, a role of immunosenescence in almost all age-related or associated diseases has been proposed including autoimmune diseases and inflammatory chronic diseases such as atherosclerosis, heart diseases and AD [73, 74].

Aging provokes a redistribution of NK cell subsets characterized by an increase of mature CD56^dim^ cells with a significant reduction of the more immature CD56^bright^ NK cell subset probably as consequence of the decreased production of bone marrow precursors in the elderly [63, 75, 76]. An increase of CD56^−^CD16^+^ NK cells is also observed in elderly donors [76]. The proportion of CD56^bright^ NK cells has been inversely correlated to C-reactive protein (CRP) levels that could be related to “inflammaging,” a condition of chronic low levels of inflammation associated with aging [77]. It has been described that both age and persistent CMV infection contribute to NK cell phenotypical and functional changes observed in the elderly. The expression of the senescence marker CD57 is increased on NK cells of elderly donors [78–80]. The accumulation of CD57+ long-lived NK cells has been associated to CMV [63].

NK cell phenotype is also altered with aging [63]. The activating natural cytotoxic receptors (NCRs) NKp46 and NKp30 and the DNAX accessory molecule-1 (DNAM-1) are decreased in the elderly [61, 79–82] whereas the expression of NKG2C activating receptor is increased [61]. Conflicting data concerning the expression of the inhibitory receptor NKG2A in elderly donors has been published. Whereas some authors found no significant differences in the expression of NKG2A on NK cells from healthy elderly donors [76, 83], another study showed a decreased expression of NKG2A [78]. The expression of KIR was shown to be maintained or increased [61, 78, 79] and the expression of NKG2D did not change with age [61].

Age induces changes in NK cell functions [63] including a decreased NK cell proliferation in response to IL-2 stimulation [84]. The increased proportion of CD56^dim^ NK cells observed with age can maintain NK cell cytotoxicity. However, an impairment of NK cell cytotoxic capacity was observed when considered on a “per cell” basis. [80, 85] probably as consequence of decreased expression of activating receptors [63, 83, 86]. Age does not provoke changes in the expression of CD16 (Fc*γ*RIII) a low affinity receptor for the Fc fragment of immunoglobulin G that is involved in antibody dependent cell cytotoxicity (ADCC) [85]. In healthy elderly individuals, the number and frequency of CD56^dim^ NK cells are increased and could contribute to the maintenance of NK cell responses against pathogens [61, 87]. Diminished NK cell function in aged individuals has been associated with an increased incidence of infections and death in elderly individuals with impaired performance status [88].

The analysis of surface receptors involved in NK cell migration showed no changes on the expression of the adhesion molecule CD2 [89] and the chemokine receptors CCR3 and CCR5 [90]. A lower surface expression of CXCR1, a receptor for IL-8, was observed on NK cells in elderly donors although the percentage of CXCR1+ NK cells was maintained [90].

Activated NK cells secrete several cytokines with immunoregulatory functions such as TNF-*α*, IFN-*γ*, IL-8, and macrophage inflammatory protein (MIP)-1*α*. These cytokines contribute to the immune response by stimulating other immune cells [91]. NK cell response to cytokines is either maintained or reduced in elderly donors compared to young individuals. The production of IFN-*γ* by unstimulated NK cells from elderly donors is impaired and can be recovered after IL-2 stimulation [83]. In response to IL-2 stimulation, an increased production of IL-1, IL-4, IL-6, IL-10, and TNF*α* by NK cells is observed in the elderly [74, 92]. Altogether, aging may lead to an altered immunoregulatory capacity of NK cells.

## 5. NK Cells and AD

The first report supporting the involvement of NK cells in the pathogenesis of AD is from Krishnaraj in 1991 showing that a drug used for the treatment of neurologic abnormalities supressed NK cell cytotoxicity [93]. As indicated above, alterations in NK cell percentage and cytotoxic activity are observed in the experimental model of triple transgenic mice for AD before the onset of AD, suggesting that changes in NK cells constitute early peripheral markers of the preclinical and prodromal stages of AD [54].

A recent study, analyses NK cell alterations in AD related syndromes and stages in comparison with age-associated changes in healthy elderly individuals [94]. The percentages of CD3−CD56+, CD56+CD16−, and CD56+CD16+ NK cells within the lymphocyte population were similar in aMCI and mild AD (mAD) patients compared to healthy individuals [94]. Likewise, another study found no differences in the number of CD56+CD16+ NK cells in AD patients [51].

Controversial results have been published regarding NK cell function in AD patients. A decreased cytotoxic function of NK cells from AD patients compared to healthy controls was reported by Araga et al. [95, 96]. In contrast, Solerte et al. demonstrated an increased production of TNF-*α* and IFN-*γ* and higher cytotoxic capacity of cytokine stimulated NK cells from AD patients compared to healthy elderly donors [97–100].

In AD patients, NK cell activity was inversely correlated with the cognitive status evaluated by the analysis of MMSE (Mini Mental State Examination) score [100]. The release of cytokines such as TNF-*α* and IFN-*γ* by NK cells has important effects on inflammation and immune responses against viral infections and tumours. In AD patients the aberrant production of these cytokines by activated NK cells has been proposed to be partially responsible of the neurodegenerative process. In addition, it has been speculated that the deposit of A*β* in the brain may constitute a feedback loop contributing to maintain the secretion of proinflammatory cytokines [97].

**Figure 2:**
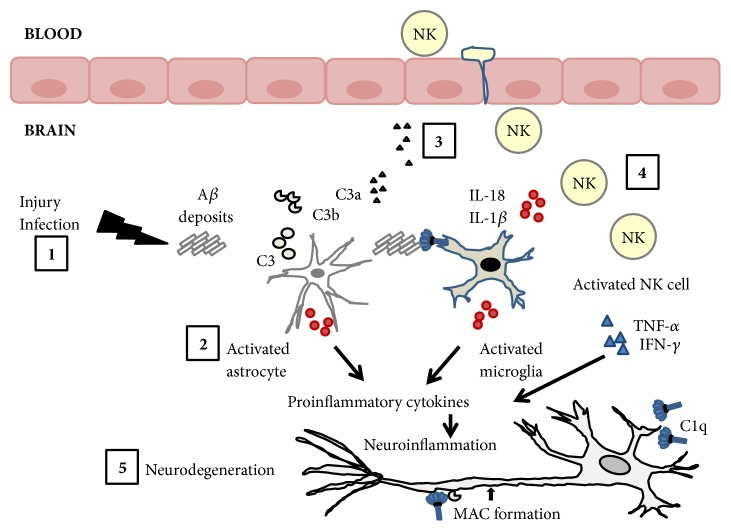
**A model of NK cell involvement in Alzheimer's disease.** Injury or infection can lead to the accumulation of A*β* peptides in the brain (1) and stimulates microglia and astrocytes (2). Accumulation of A*β* forming plaques induces the activation of complement system. C1q secreted by neurons can bind A*β* and activates C1q receptor (C1qR) on microglia promoting phagocytosis of A*β*. In addition, activated microglia secretes the proinflammatory cytokines IL-1*β* and IL-18.Astrocytes stimulated by inflammatory signals secrete C3 that is cleaved into C3b and C3a. C3a mediates recruitment of immune cells. This inflammatory environment can support trafficking of peripheral NK cells into the brain (3). NK cells are activated by microglia secreted cytokines and produce IFN-*γ* and TNF-*α* (4). Complement activation and proinflammatory cytokines can lead to neuronal damage and death (5).

NK cell interaction with cerebral innate immune cells such as microglia and astrocytes has been described. These cells protect the brain from insults such as infections and injury and can regulate the inflammatory response. In the brain, cytokine and chemokine responses after an insult have a relevant role recruiting circulating lymphoid cells including NK cells (Figure 2) and myeloid cells that further sustain immune responses in the brain [101].

The analysis of NK cell subsets in healthy elderly individuals, aMCI and mAD patients showed no significant differences among these three groups [94]. Regarding the expression of activating and inhibitory receptors, it was shown that the expression of CD57, a marker of terminally differentiated NK cells, NKG2D, and CD94, was not altered on NK cells from aMCI and mAD patients compared to elderly healthy donors. In contrast, the expression of NKG2A is reduced in aMCI [94]. The decreased expression of NKG2A could contribute to NK cell activation in aMCI patients compared to mAD. CD16 expression was increased in mAD patients but not in aMCI subjects although its relevance in AD remains to be determined [94].

Human NK cells express TLR2 and TLR4 receptors that recognize A*β* [45, 46]. Whereas no changes in the expression of TLR4 was observed, TLR2 expression was significantly lower in NK cells from mAD patients compared to healthy controls. The expression of TLR9, a receptor for unmethylated CpG on DNA, is reduced in mAD subjects compared to healthy controls and aMCI patients suggesting a role in AD progression [94].

NK cell cytotoxic function is preserved in aMCI and mAD patients. Thus, i*n vitro* assays showed that activated NK cells from aMCI and mAD patients lyse K562 cells, an erythroleukemia cell line [94]. Interestingly, the expression of granzyme B and CD95 was increased in aMCI patients compared to healthy individuals and mAD patients [94]. Further studies are required to establish if these increases represent NK cell activation in response to pathogens or other insults in aMCI patients.

The involvement of NK cells in inflammation and neuroinflammation in AD has been suggested [74]. Although an increase in the expression of IL-18R*β* has been shown in T cells of aMCI and AD patients [102], neither IL-18R*β* nor IL-12R*β*1/*β*2 were altered on NK cells of aMCI and mAD patients compared to healthy elderly controls [94].

NK cells from AD patients upregulate cytokine production* in vitro* in response to IL-2 [97]. The production of TNF-*α* and IFN-*γ* by NK cells stimulated with IL-12 and K562 cells was increased in aMCI patients compared to healthy donors and mAD patients [94].

Chemokine receptors are differentially expressed in NK subsets and play a pivotal role in NK cell migration [103]. CX3CR1, a receptor for CX3CL1 (fractalkine) is expressed on CD56^dim^CD16^+^; NK cells has been linked to NK cell migration to the brain in patients with multiple sclerosis [104]. However, the expression of CX3CR1 and CCR5 on NK cells from aMCI and AD patients was similar to healthy donors [94]. CCR7 is a chemokine receptor for CCL19 and CCL21 that is involved in the homing of immune cells to secondary lymphoid organs. The expression of CCR7 on NK cells was found upregulated in aMCI compared to healthy donors [94]. Interestingly,* in vitro* analysis of CCR7 mediated chemotaxis showed a decrease in CCL19-induced chemotaxis of NK cells from aMCI and AD patients compared to healthy donors [94].

## 6. Conclusions

In conclusion, despite the recent findings concerning the role of NK cells in AD development and pathogenesis more efforts are required to further characterize NK cells according to the expression of activating and inhibitory receptors in AD patients. The pattern of expression of chemokine receptors involved in NK cell migration together with the activation status of NK cells in these patients may constitute biomarkers of AD progression and open new possibilities for treatment directed to NK cells. In addition the role of persistent viral infections such as CMV in AD and its effect on NK cells should be further analysed.

## References

[1] Scheltens P., Blennow K., Breteler M. M. (2016). Alzheimer's disease. *The Lancet*.

[2] Prince M., Bryce R., Albanese E., Wimo A., Ribeiro W., Ferri C. P. (2013). The global prevalence of dementia: a systematic review and metaanalysis. *Alzheimer’s & Dementia*.

[3] Sosa-Ortiz A. L., Acosta-Castillo I., Prince M. J. (2012). Epidemiology of Dementias and Alzheimer's Disease. *Archives of Medical Research*.

[4] Alzheimer's Association (2018). 2018 Alzheimers disease facts and figures. *Alzheimer’s & Dementia*.

[5] Sadigh-Eteghad S., Sabermarouf B., Majdi A., Talebi M., Farhoudi M., Mahmoudi J. (2015). Amyloid-beta: a crucial factor in Alzheimer's disease. *Medical Principles and Practice*.

[6] De-Paula V. J., Radanovic M., Diniz B. S., Forlenza O. V., Harris J. (2012). Alzheimer’s disease. *Protein Aggregation and Fibrillogenesis in Cerebral and Systemic Amyloid Disease*.

[7] McGeer P. L., McGeer E. G. (2013). The amyloid cascade-inflammatory hypothesis of Alzheimer disease: implications for therapy. *Acta Neuropathologica*.

[8] Fulop T., Lacombe G., Cunnane S. (2013). Elusive alzheimer's disease: Can immune signatures help our understanding of this challenging disease? Part 2: New immune paradigm. *Discovery Medicine*.

[9] Fulop T., Lacombe G., Cunnane S. (2013). Elusive alzheimer's disease: Can immune signatures help our understanding of this challenging disease? Part 1: Clinical and historical background. *Discovery Medicine*.

[10] Dá Mesquita S., Ferreira A. C., Sousa J. C., Correia-Neves M., Sousa N., Marques F. (2016). Insights on the pathophysiology of Alzheimer's disease: the crosstalk between amyloid pathology, neuroinflammation and the peripheral immune system. *Neuroscience & Biobehavioral Reviews*.

[11] Van Eldik L. J., Carrillo M. C., Cole P. E. (2016). The roles of inflammation and immune mechanisms in Alzheimer's disease. *Alzheimer's and Dementia: Translational Research and Clinical Interventions*.

[12] McManus R. M., Heneka M. T. (2017). Role of neuroinflammation in neurodegeneration: New insights. *Alzheimer’s Research & Therapy*.

[13] VanItallie T. B. (2017). Alzheimer's disease: Innate immunity gone awry?. *Metabolism - Clinical and Experimental*.

[14] Blasko I., Grubeck-Loebenstein B. (2003). Role of the immune system in the pathogenesis, prevention and treatment of Alzheimer's disease. *Drugs & Aging*.

[15] Le Page A., Dupuis G., Frost E. H. (2018). Role of the peripheral innate immune system in the development of Alzheimer's disease. *Experimental Gerontology*.

[16] Small D. H., Cappai R. (2006). Alois Alzheimer and Alzheimer's disease: A centennial perspective. *Journal of Neurochemistry*.

[17] Albert M. S., DeKosky S. T., Dickson D. (2011). The diagnosis of mild cognitive impairment due to Alzheimer's disease: recommendations from the National Institute on Aging-Alzheimer's Association workgroups on diagnostic guidelines for Alzheimer's disease. *Alzheimer’s & Dementia*.

[18] McKhann G. M., Knopman D. S., Chertkow H. (2011). The diagnosis of dementia due to Alzheimer's disease: Recommendations from the National Institute on Aging-Alzheimer's Association workgroups on diagnostic guidelines for Alzheimer's disease. *Alzheimer’s & Dementia*.

[19] Jack C. R., Albert M. S., Knopman D. S. (2011). Introduction to the recommendations from the National Institute on Aging-Alzheimer's Association workgroups on diagnostic guidelines for Alzheimer's disease. *Alzheimer’s & Dementia*.

[20] Jack C. R., Bennett D. A., Blennow K. (2016). A/T/N: An unbiased descriptive classification scheme for Alzheimer disease biomarkers. *Neurology*.

[21] Aisen P. S., Cummings J., Jack Jr C. R. (2017). On the path to 2025: understanding the Alzheimer’s disease continuum. *Alzheimer's Research & Therapy*.

[22] Frisoni G. B., Boccardi M., Barkhof F. (2017). Strategic roadmap for an early diagnosis of Alzheimer's disease based on biomarkers. *The Lancet Neurology*.

[23] Petersen R. C., Roberts R. O., Knopman D. S. (2009). Mild cognitive impairment: ten years later. *JAMA Neurology*.

[24] Ekman U., Ferreira D., Westman E. (2018). The A/T/N biomarker scheme and patterns of brain atrophy assessed in mild cognitive impairment. *Scientific Reports*.

[25] Hebert L. E., Weuve J., Scherr P. A., Evans D. A. (2013). Alzheimer disease in the United States (2010–2050) estimated using the 2010 census. *Neurology*.

[26] Holmes C., Cotterell D. (2009). Role of infection in the pathogenesis of Alzheimer's disease: Implications for treatment. *CNS Drugs*.

[27] Itzhaki R. F., Lathe R., Balin B. J. (2016). Microbes and Alzheimer's disease. *Journal of Alzheimer's Disease*.

[28] Miklossy J. (2011). Emerging roles of pathogens in Alzheimer disease. *Expert Reviews in Molecular Medicine*.

[29] Hogestyn J. M., Mock D. J., Mayer-Proschel M. (2018). Contributions of neurotropic human herpesviruses herpes simplex virus 1 and human herpesvirus 6 to neurodegenerative disease pathology. *Neural Regeneration Research*.

[30] Agostini S., Mancuso R., Baglio F. (2016). Lack of Evidence for a Role of HHV-6 in the Pathogenesis of Alzheimer's Disease. *Journal of Alzheimer's Disease*.

[31] Carbone I., Lazzarotto T., Ianni M. (2014). Herpes virus in alzheimer's disease: Relation to progression of the disease. *Neurobiology of Aging*.

[32] Readhead B., Haure-Mirande J. V., Funk C. C. (2018). Multiscale analysis of independent alzheimer's cohorts finds disruption of molecular, genetic, and clinical networks by human herpesvirus. *Neuron*.

[33] Holmes C., Cunningham C., Zotova E. (2009). Systemic inflammation and disease progression in Alzheimer disease.. *Neurology*.

[34] Lövheim H., Olsson J., Weidung B. (2018). Interaction between cytomegalovirus and herpes simplex virus type 1 associated with the risk of alzheimer's disease development. *Journal of Alzheimer's Disease*.

[35] Ballard C., Corbett A. (2013). Agitation and aggression in people with Alzheimer's disease. *Current Opinion in Psychiatry*.

[36] Sun X., Chen W. D., Wang Y. D. (2015). Beta-Amyloid: the key peptide in the pathogenesis of Alzheimer's disease. *Frontiers in Pharmacology*.

[37] Hanger D. P., Lau D. H. W., Phillips E. C. (2014). Intracellular and extracellular roles for tau in neurodegenerative disease. *Journal of Alzheimer's Disease*.

[38] Dansokho C., Heneka M. T. (2018). Neuroinflammatory responses in Alzheimer's disease. *Journal of Neural Transmission*.

[39] Spangenberg E. E., Green K. N. (2017). Inflammation in Alzheimer's disease: lessons learned from microglia-depletion models. *Brain, Behavior, and Immunity*.

[40] Sarlus H., Heneka M. T. (2017). Microglia in Alzheimer's disease. *The Journal of Clinical Investigation*.

[41] Jevtic S., Sengar A. S., Salter M. W., McLaurin J. (2017). The role of the immune system in Alzheimer disease: Etiology and treatment. *Ageing Research Reviews*.

[42] Tan M.-S., Yu J.-T., Jiang T., Zhu X.-C., Tan L. (2013). The NLRP3 inflammasome in Alzheimer's disease. *Molecular Neurobiology*.

[43] Zhou Keren, Shi Ligen, Wang Yan, Chen Sheng, Zhang Jianmin (2016). Recent Advances of the NLRP3 Inflammasome in Central Nervous System Disorders. *Journal of Immunology Research*.

[44] Della Chiesa M., Marcenaro E., Sivori S., Carlomagno S., Pesce S., Moretta A. (2014). Human NK cell response to pathogens. *Seminars in Immunology*.

[45] Liu S., Liu Y., Hao W. (2012). TLR2 is a primary receptor for Alzheimer's amyloid beta peptide to trigger neuroinflammatory activation. *The Journal of Immunology*.

[46] Udan M. L. D., Ajit D., Crouse N. R., Nichols M. R. (2008). Toll-like receptors 2 and 4 mediate A*β*(1-42) activation of the innate immune response in a human monocytic cell line. *Journal of Neurochemistry*.

[47] Molteni M., Rossetti C. (2017). Neurodegenerative diseases: the immunological perspective. *Journal of Neuroimmunology*.

[48] Petersen A. M. W., Pedersen B. K. (2005). The anti-inflammatory effect of exercise. *Journal of Applied Physiology*.

[49] Petersen A. M., Pedersen B. K. (2006). The role of IL-6 in mediating the anti-inflammatory effects of exercise. *Journal of Physiology and Pharmacology*.

[50] Pellicanò M., Larbi A., Goldeck D. (2012). Immune profiling of Alzheimer patients. *Journal of Neuroimmunology*.

[51] Richartz-Salzburger E., Batra A., Stransky E. (2007). Altered lymphocyte distribution in Alzheimer's disease. *Journal of Psychiatric Research*.

[52] Speciale L., Calabrese E., Saresella M. (2007). Lymphocyte subset patterns and cytokine production in Alzheimer's disease patients. *Neurobiology of Aging*.

[53] Poli A., Kmiecik J., Domingues O. (2013). NK cells in central nervous system disorders. *The Journal of Immunology*.

[54] Maté I., Cruces J., Giménez-Llort L., De La Fuente M. (2014). Function and redox state of peritoneal leukocytes as preclinical and prodromic markers in a longitudinal study of triple-transgenic mice for Alzheimer's disease. *Journal of Alzheimer's Disease*.

[55] Spits H., Bernink J. H., Lanier L. (2016). NK cells and type 1 innate lymphoid cells: Partners in host defense. *Nature Immunology*.

[56] Cooper M. A., Fehniger T. A., Caligiuri M. A. (2001). The biology of human natural killer-cell subsets. *Trends in Immunology*.

[57] Fauriat C., Long E. O., Ljunggren H.-G., Bryceson Y. T. (2010). Regulation of human NK-cell cytokine and chemokine production by target cell recognition. *Blood*.

[58] Ouyang Q., Baerlocher G., Vulto I., Lansdorp P. M. (2007). Telomere length in human natural killer cell subsets. *Annals of the New York Academy of Sciences*.

[59] Tarazona R., Casado J. G., Delarosa O. (2002). Selective depletion of CD56(dim) NK cell subsets and maintenance of CD56(bright) NK cells in treatment-naive HIV-1-seropositive individuals. *Journal of Clinical Immunology*.

[60] Kared H., Martelli S., Ng T. P., Pender S. L. F., Larbi A. (2016). CD57 in human natural killer cells and T-lymphocytes. *Cancer Immunology, Immunotherapy*.

[61] Gayoso I., Sanchez-Correa B., Campos C. (2011). Immunosenescence of human natural killer cells. *Journal of Innate Immunity*.

[62] Cichocki F., Miller J. S., Anderson S. K., Bryceson Y. T. (2013). Epigenetic regulation of NK cell differentiation and effector functions. *Frontiers in Immunology*.

[63] Solana R., Campos C., Pera A., Tarazona R. (2014). Shaping of NK cell subsets by aging. *Current Opinion in Immunology*.

[64] Tesi B., Schlums H., Cichocki F., Bryceson Y. T. (2016). Epigenetic Regulation of Adaptive NK Cell Diversification. *Trends in Immunology*.

[65] Long E. O., Sik Kim H., Liu D., Peterson M. E., Rajagopalan S. (2013). Controlling natural killer cell responses: integration of signals for activation and inhibition. *Annual Review of Immunology*.

[66] Narni-Mancinelli E., Ugolini S., Vivier E. (2013). Tuning the threshold of natural killer cell responses. *Current Opinion in Immunology*.

[67] Tarazona R., Gayoso I., Alonso C., Fulop T., Franceschi C., Hirokawa K., Pawelec G. (2009). NK cells in human ageing. *Handbook on Immunosenescence*.

[68] Moretta L., Montaldo E., Vacca P. (2014). Human natural killer cells: Origin, receptors, function, and clinical applications. *International Archives of Allergy and Immunology*.

[69] Tarazona R., Sanchez-Correa B., Casas-Aviles I. (2017). Immunosenescence: limitations of natural killer cell-based cancer immunotherapy. *Cancer Immunology, Immunotherapy*.

[70] Chan C. J., Smyth M. J., Martinet L. (2014). Molecular mechanisms of natural killer cell activation in response to cellular stress. *Cell Death & Differentiation*.

[71] López-Otín C., Blasco M. A., Partridge L., Serrano M., Kroemer G. (2013). The hallmarks of aging. *Cell*.

[72] Wikby A., Nilsson B.-O., Forsey R. (2006). The immune risk phenotype is associated with IL-6 in the terminal decline stage: Findings from the Swedish NONA immune longitudinal study of very late life functioning. *Mechanisms of Ageing and Development*.

[73] Martorana A., Bulati M., Buffa S. (2012). Immunosenescence, inflammation and Alzheimer's disease. *Longevity & Healthspan*.

[74] Camous Xavier, Pera Alejandra, Solana Rafael, Larbi Anis (2012). NK Cells in Healthy Aging and Age-Associated Diseases. *Journal of Biomedicine and Biotechnology*.

[75] Chidrawar S. M., Khan N., Chan Y. L., Nayak L., Moss P. A. (2006). Ageing is associated with a decline in peripheral blood CD56bright NK cells. *Immunity & Ageing*.

[76] Campos C., Pera A., Sanchez-Correa B. (2014). Effect of age and CMV on NK cell subpopulations. *Experimental Gerontology*.

[77] Campos C., Pera A., Lopez-Fernandez I., Alonso C., Tarazona R., Solana R. (2014). Proinflammatory status influences NK cells subsets in the elderly. *Immunology Letters*.

[78] Lutz C. T., Moore M. B., Bradley S., Shelton B. J., Lutgendorf S. K. (2005). Reciprocal age related change in natural killer cell receptors for MHC class I. *Mechanisms of Ageing and Development*.

[79] Almeida-Oliveira A., Smith-Carvalho M., Porto L. C. (2011). Age-related changes in natural killer cell receptors from childhood through old age. *Human Immunology*.

[80] Hazeldine J., Hampson P., Lord J. M. (2012). Reduced release and binding of perforin at the immunological synapse underlies the age-related decline in natural killer cell cytotoxicity. *Aging Cell*.

[81] Sanchez-Correa B., Gayoso I., Bergua J. M. (2012). Decreased expression of DNAM-1 on NK cells from acute myeloid leukemia patients. *Immunology & Cell Biology*.

[82] Sanchez-Correa B., Morgado S., Gayoso I. (2011). Human NK cells in acute myeloid leukaemia patients: Analysis of NK cell-activating receptors and their ligands. *Cancer Immunology, Immunotherapy*.

[83] Le Garff-Tavernier M., Béziat V., Decocq J. (2010). Human NK cells display major phenotypic and functional changes over the life span. *Aging Cell*.

[84] Borrego F., Alonso M. C., Galiani M. D. (1999). NK phenotypic markers and IL2 response in NK cells from elderly people. *Experimental Gerontology*.

[85] Solana R., Mariani E. (2000). NK and NK/T cells in human senescence. *Vaccine*.

[86] Solana R., Tarazona R., Gayoso I., Lesur O., Dupuis G., Fulop T. (2012). Innate immunosenescence: effect of aging on cells and receptors of the innate immune system in humans. *Seminars in Immunology*.

[87] Solana R., Alonso M. C., Peña J. (1999). Natural killer cells in healthy aging. *Experimental Gerontology*.

[88] Ogata K., An E., Shioi Y. (2001). Association between natural killer cell activity and infection in immunologically normal elderly people. *Clinical & Experimental Immunology*.

[89] Hayhoe R. P. G., Henson S. M., Akbar A. N., Palmer D. B. (2010). Variation of human natural killer cell phenotypes with age: identification of a unique KLRG1-negative subset. *Human Immunology*.

[90] Mariani E., Meneghetti A., Neri S. (2002). Chemokine production by natural killer cells from nonagenarians. *European Journal of Immunology*.

[91] Hazeldine J., Lord J. M. (2013). The impact of ageing on natural killer cell function and potential consequences for health in older adults. *Ageing Research Reviews*.

[92] Rink L., Cakman I., Kirchner H. (1998). Altered cytokine production in the elderly. *Mechanisms of Ageing and Development*.

[93] Krishnaraj R. (1991). Immunomodulation by 9-amino-1,2,3,4-tetrahydroacridine (THA): 1. Down-regulation of natural cell-mediated cytotoxicity in vitro. *International Journal of immunopharmacology*.

[94] Le Page A., Bourgade K., Lamoureux J. (2015). NK cells are activated in amnestic mild cognitive impairment but not in mild Alzheimer's disease patients. *Journal of Alzheimer's Disease*.

[95] Araga S., Kagimoto H., Funamoto K., Adachi A., Inoue K., Takahashi K. (1990). Natural Killer Cell Activity in Patients With Dementia of the Alzheimer Type. *JAMA Neurology*.

[96] Araga S., Kagimoto H., Funamoto K., Takahashi K. (1991). Reduced natural killer cell activity in patients with dementia of the Alzheimer type. *Acta Neurologica Scandinavica*.

[97] Solerte S. B., Cravello L., Ferrari E., Fioravanti M. (2000). Overproduction of IFN-*γ* and TNF-*α* from natural killer (NK) cells is associated with abnormal NK reactivity and cognitive derangement in Alzheimer's disease. *Annals of the New York Academy of Sciences*.

[98] Solerte S. B., Fioravanti M., Severgnini S. (1996). Enhanced cytotoxic response of natural killer cells to lnterleukin-2 in alzheimer’s disease. *Dementia and Geriatric Cognitive Disorders*.

[99] Solerte S. B., Ceresini G., Ferrari E., Fioravanti M. (2000). Hemorheological changes and overproduction of cytokines from immune cells in mild to moderate dementia of the Alzheimer's type: adverse effects on cerebromicrovascular system. *Neurobiology of Aging*.

[100] Solerte S. B., Fioravanti M., Pascale A., Ferrari E., Govoni S., Battaini F. (1998). Increased natural killer cell cytotoxicity in Alzheimer's disease may involve protein kinase C dysregulation. *Neurobiology of Aging*.

[101] Ransohoff R. M., Brown M. A. (2012). Innate immunity in the central nervous system. *The Journal of Clinical Investigation*.

[102] Salani F., Ciaramella A., Bizzoni F. (2013). Increased expression of Interleukin-18 receptor in blood cells of subjects with Mild Cognitive Impairment and Alzheimer's disease. *Cytokine*.

[103] Bernardini G., Gismondi A., Santoni A. (2012). Chemokines and NK cells: regulators of development, trafficking and functions. *Immunology Letters*.

[104] Infante-Duarte C., Weber A., Krätzschmar J. (2005). Frequency of blood CX3CR1-positive natural killer cells correlates with disease activity in multiple sclerosis patients. *The FASEB Journal*.

